# The role of age-specific N-terminal pro-brain natriuretic peptide cutoff values in predicting intravenous immunoglobulin resistance in Kawasaki disease: a prospective cohort study

**DOI:** 10.1186/s12969-019-0368-8

**Published:** 2019-09-18

**Authors:** Shuran Shao, Chunyan Luo, Kaiyu Zhou, Yimin Hua, Mei Wu, Lei Liu, Xiaoliang Liu, Chuan Wang

**Affiliations:** 1 Department of Pediatric Cardiology, West China Second University Hospital, Sichuan University, No. 20, 3rd section, South Renmin Road Chengdu, Chengdu, 610041 Sichuan China; 2 The Cardiac development and early intervention unit, West China Institute of Women and Children’s Health, West China Second University Hospital, Sichuan University, Chengdu, Sichuan China; 3 Department of Radiology, West China Hospital, Sichuan University, Chengdu, Sichuan China; 4 Key Laboratory of Birth Defects and Related Diseases of Women and Children (Sichuan University), Ministry of Education, Chengdu, Sichuan China; 5 Key Laboratory of Development and Diseases of Women and Children of Sichuan Province, West China Second University Hospital, Sichuan University, Chengdu, Sichuan China

**Keywords:** Kawasaki disease, N-terminal pro-brain natriuretic peptide, Age-stratified, Intravenous immunoglobulin resistance

## Abstract

**Background:**

The prediction of resistance to intravenous immunoglobulins (IVIG) is currently still one of the main research areas in Kawasaki disease (KD). Several studies have reported on the use of N-terminal pro-brain natriuretic peptide (NT-ProBNP) to this end. However, considering the age-dependency of NT-ProBNP levels, age- specific NT-ProBNP cutoff levels to predict IVIG resistance in KD might be more precise and should be evaluated.

**Methods:**

A prospective cohort study with standardized data collection involving 393 KD patients aged 1 month to 125 months was conducted between June 2015 and April 2018. The demographic characteristics, clinical manifestations and laboratory data were compared between the patients responding to initial intravenous immunoglobulin (IVIG-response group) and those who did not (IVIG-resistance group). We further distinguished four subgroups according to patients’ age (< 1 year, 1–2 years, 2–6 years, > 6 years). The cutoff values of NT-ProBNP for the prediction of IVIG resistance overall and in the subgroups were obtained using receiver operating characteristic (ROC) analysis.

**Results:**

In all KD patients, the level of NT-ProBNP was significantly higher in the IVIG-resistance compared to the IVIG-response group (*P* = 0.006). This findings was similar in the subgroups except for patients older than six years. The best cutoff values of NT-ProBNP to predict IVIG resistance were 3755 pg/ml for all KD patients, 3710 pg/ml, 2800 pg/ml, 2480 pg/ml for those aged 2–6 years, 1–2 years and < 1 year, respectively. The corresponding sensitivities were 44.0, 52.2, 50.0 and 75.0%, while the specifities were 84.1, 86.3, 77.9 and 71.8%, respectively.

**Conclusions:**

NT-proBNP is a complementary laboratory marker for the prediction of IVIG resistance in KD patients, particularly for those younger than one year. Applying age-specific cutoff values is more precise than one value for all ages.

## Background

Kawasaki Disease (KD) is an acute general vasculitis of unknown etiology that mainly occurrs in infants and children under five years of age. While the timely initiation of treatment with intravenous immunoglobulin (IVIG) can effectively reduce the development of coronary artery lesions (CALs), approximately 10–20% of patients do not respond to IVIG treatment, and have a higher risk of CALs [1]. Thus, it is critical and clinically significant to identify these patients before initial IVIG treatment, because they may benefit from more aggressive therapy such as corticosteroid [2], monoclonal antibodies [3–5], cytotoxic agents [6], or plasma exchange [7].

The levels of N-terminal ProBrain Natriuretic Peptide (NT-ProBNP), cleaved by ProBNP, increase in ventricles dysfunction and wall stress [8]. Several studies have verified that NT-ProBNP is a sensitive biomarker of congestive heart failure and acute myocardial infarction [9–13]. Furthermore, the importance of NT-ProBNP in the prediction of IVIG resistance in KD has also been shown in studies [14–17]. The study performed by Kim [14] concluded that NT-ProBNP≥1093 pg/ml might predict IVIG resistance, while Yoshimura [16] reported that NT-ProBNP≥800 pg/ml might predict IVIG resistance in a Japanese population. Another research conducted by Kim et al. [15] in Korea, suggested that NT-proBNP≥479 pg/ml was a useful marker for IVIG resistance, whereas Lee et al. [17] found that NT-proBNP≥628.6 pg/ml might predict IVIG resistance. However, most of these studies were limited by their small sample size (*n* = 80 [16], *n* = 129 [15], and *n* = 135 [14]) and some of them [14, 17] were of a retrospective design. Most importantly, however, the normal range of NT-proBNP varies widely with age [18–21]. Therefore, applying the same cutoff value for NT-proBNP to patients regardless of their age would be unreasonable. We performed a prospective cohort study in an appropriately large sample to assess the effectiveness of age-specific NT-proBN*P* values in predicting IVIG resistance in KD and to determine the best cutoff values of NT-ProBNP for different age groups.

## Methods

We prospectively recruited patients with KD who were hospitalized at the Department of Pediatrics of the West China Second University Hospital of Sichuan University (WCSUH-SCU), which is the largest medical center for children in Southwest China, between June 2015 and April 2018. The diagnosis of KD relied on standards recommended by the American Heart Association’s scientific statement for diagnosis, treatment, and long-term management of KD [22], and was e confirmed by two experienced pediatricians (at least one of them is a KD specialist). Structured questionnaires with pre-coded questions including basic demographic information, clinical manifestations, hematological examination results, treatment and follow up outcomes, were used for data collection. All questionnaires were pretested and revised accordingly. Two well-trained physicians conducted the data collection. The questionnaires were double-checked to assure their completeness.

Informed written consent for the use of the obtained data was obtained from the parents after the nature of this study had been fully explained to them. The study was approved by the University Ethics Committee on Human Subjects at Sichuan University.

In total, 540 patients were diagnosed with KD on admission during the period of the study. Patients who had received initial IVIG treatment at other medical facilities (*n* = 74) or did not receive IVIG treatment between four and ten days from fever onset (*n* = 20) were excluded. Another 30 patients were excluded because IVIG treatment had been initiated before blood sampling. Additionally, we excluded 23 patients because of incomplete laboratory data (*n* = 16) or lack of follow-up results (n = 7). Finally, the data of 393 patients was analyzed. Of these, seven suffered from KD shock syndrome (KDSS).

Serum samples were obtained to measure serum NT-proBNP levels using an electrochemiluminescence immunoassay (Roche Diagnostics, Germany) on the day that IVIG was started. At the same time, other laboratory parameters were also obtained and analyzed. Due to the assay-dependent of NT-ProBNP detection, the age-group stratification was based on a previous study [18], which presented a summary of four studies that measured NT-ProBNP levels in normal infants and children using the Roche assay. In that article [18], the normal values of NT-ProBNP in children aged 0–2 days (median, 3183 pg/ml, range, 260-13,224 pg/ml), 3–11 days (median, 2210 pg/ml, range, 28-7250 pg/ml), 1 month-1 year (median, 141 pg/ml, range, 5-1121 pg/ml), 1–2 years (median, 129 pg/ml, range, 31-675 pg/ml), 2–6 years (median, 70 pg/ml, range, 5-391 pg/ml), 6–14 years (median, 52 pg/ml, range, 5-391 pg/ml), and 14–18 years (median, 34 pg/ml, range, 5-363 pg/ml) were shown. Since the youngest child in our study population was one month and only a small number of subjects were older than 6 years, we ultimately classified study participants into four groups: < 1 year [*n* = 79, 20.1%], 1–2 years [*n* = 109, 27.7%], 2–6 years [*n* = 176, 44.8%], and > 6 years [*n* = 29, 7.4%].

All patients received 2 g/kg of IVIG for 24 h and 30–50 mg/kg/day of aspirin until they were afebrile. A negative response to initial treatment with IVIG was defined as a fever over 36 h after the end of the IVIG infusion or recurrent fever with evidence of systemic inflammation after an afebrile period [22]. Of the 393 patients, 54 patients who were resistant to the initial IVIG received a second IVIG dose (1 g/kg). Of these, 32 patients responded to the second dose, and the remaining 22 patients were treated with high doses of methylprednisolone (10-30 mg/kg).

The definition of a CAL is that the internal diameter of the coronary artery exceeds 3 mm in a child younger than five years, 4 mm for children for five years and older, or an internal segment with a diameter that is at least 1.5 times wider than the diameter of the adjacent segment, or if the lumen appears irregular [23]. According to our institutional standard protocol, patients underwent standardized echocardiography by two pediatric ultrasonic experts before initial treatment, and ultrasound was repeated every two weeks to eight weeks later in the cardiology clinic follow-up evaluations until the CALs had resolved.

The patients were categorized into two groups according to whether they responded to the initial IVIG treatment: those who respond to the initial IVIG treatment (IVIG-response group), and those who resisted to the initial IVIG treatment (IVIG-resistance group), and also whether they were complicated with CAL: those who developed a significant CAL (CAL group) and those who did not develop (non-CAL group).

### Statistical analysis

Data analysis was performed with SPSS 17.0 (SPSS Inc. Chicago, IL, USA). Quantitative data are presented as the median with the 25th and 75th percentiles (interquartile range (IQR)) in square brackets, while qualitative data are expressed as the number (n) and percentage (%) as appropriate. The shapiro-Wilk test and homogeneity test of variance were used to confirm that quantitative data from different groups were normally distributed and met the criteria for homogeneity of variance. The chi-square and unpaired Student’s t-test/ Mann–Whitney U test were applied to compare the demographic characteristics, clinical manifestations and laboratory data between the IVIG-response and IVIG-resistance group. The cutoff values of NT-ProBNP for predicting IVIG resistance were obtained using receiver operating characteristic (ROC) analysis. *P*-values < 0.05 were considered to be statistically significant.

## Results

Table 1 shows the comparison of the demographic characteristics, clinical manifestation and laboratory data between the IVIG-response and IVIG-resistance group. The nonresponders and responders did not differ significantly in terms of age, gender, fever duration at the initial treatment, typical clinical manifestations of KD, or the mean time from fever onset to the blood test (all *P* > 0.05). The frequency of cardiac abnormalities showed no difference between the two groups except for pericardial effusion (*P* = 0.006). Nonresponders had a higher neutrophil ratio (*P* = 0.003), C-reactive protein (CRP) (*P* = 0.022) and total bilirubin level (*P* = 0.018), and a lower platelet count (*P* < 0.001), albumin (*P* = 0.002), serum sodium (P < 0.001) and potassium level (*P* = 0.026).

**Table 1:** Comparison of the demographic characteristics, clinical and laboratory data between the IVIG-response and IVIG-resistance patients with KD in total age before initial IVIG treatment

	IVIG-resistance (*n* = 54)	IVIG-response (*n* = 339)	P value
Age (months)	28.50 [14.00–57.00]	24.00 [13.00–42.00]	0.051
Male (%)	28(51.9)	199(58.7)	0.344
Clinical manifestations			
Rash, n (%)	46(85.2)	263(77.6)	0.206
Bilateral bulbar conjunctive injection, n (%)	48(88.9)	312(92.0)	0.430
Edema & erythema of the extremities, n (%)	33(61.1)	208(61.4)	0.973
Erythema of oral and pharyngeal mucosa, n (%)	53(98.1)	317(93.5)	0.343
Cervical lymphadenopathy, n (%)	29(53.7)	152(44.8)	0.225
Incomplete KD, n (%)	15(27.8)	117(34.5)	0.330
Pericardial effusion (%)	6(11.1)	8(2.4)	0.006*
Valve regurgitation (%)	9(16.7)	37(1.9)	0.222
Cardiac enlargement (%)	7(13.0)	30(8.8)	0.336
Ventricular systolic dysfunction (%)	1(1.9)	1(0.3)	0.256
Coronary artery lesions (CALs), n (%)	10(18.5)	35(10.3)	0.079
Blood test from fever onset, days	5.00 [4.00–5.00]	5.00 [4.00–5.00]	0.076
Fever duration before IVIG administration, days	5.00 [5.00–6.00]	5.00 [5.00–6.00]	0.116
Laboratory features			
WBC count (10^9^/L)	14.15 [10.83–16.50]	13.40 [10.60–16.70]	0.863
Neutrophils (%)	71.30 [61.15–83.93]	66.20 [56.00–76.20]	0.003*
Hemoglobin (g/L)	106.50 [97.75–115.00]	108.00 [101.00–115.00]	0.553
PLT count (10^9^/L)	294.50 [239.25–343.75]	330.00 [276.00–404.00]	<.001*
CRP (mg/L)	85.00 [61.75–137.50]	69.00 [41.00–103.00]	0.022*
ESR (mm/h)	66.00 [45.50–94.00]	64.00 [47.00–81.00]	0.443
AST (IU/L)	30.50 [23.00–57.50]	30.00 [24.00–47.00]	0.896
ALT (IU/L)	44.00 [25.75–96.50]	36.00 [20.00–74.00]	0.809
ALB (g/L)	36.05 [32.00–38.90]	37.60 [35.20–41.10]	0.002*
Total bilirubin (mg/L)	6.80 [4.75–12.90]	6.10 [3.70–8.70]	0.018*
Urea nitrogen (mmol/L)	2.90 [2.40–3.50]	2.70 [2.10–3.20]	0.063
Creatinine (umol/L)	29.00 [24.00–36.00]	27.00 [22.00–31.00]	0.123
Sodium (mmol/L)	135.00 [132.75–137.00]	137.00 [135.00–139.00]	< 0.001*
Potassium (mmol/L)	4.04 [3.50–4.41]	4.12 [3.77–4.56]	0.026*
Troponin (ug/L)	0.12 [0.12–0.13]	0.12 [0.12–0.12]	0.139

The data are presented as the median with the 25th and 75th percentiles in square brackets for continuous variables and as the percentage for the categorical variables

IVIG, intravenous immunoglobulin; CALs, Coronary artery lesions; WBC, white blood cell; PLT, platelet; ESR, erythrocyte sedimentation rate; CRP, C-reactive protein; AST, aspartate aminotransferase; ALT, alanine aminotransferase; ALB, Albumin; NT-ProBNP, N-terminal probrain natriuretic peptide; *Statistically significant (*P* < 0.05)

**Fig. 1:**
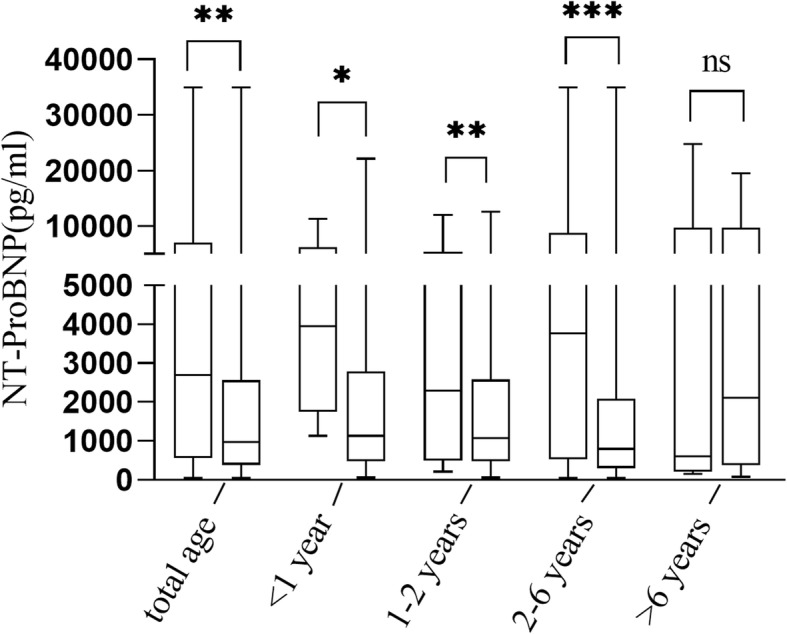
Comparsion of N-terminal pro-brain natriuretic peptide level between IVIG-resistant and IVIG-response group stratified by age. *p < 0.05; **p < 0.01; ***p < 0.001

As shown in Table 2 and Fig. 1, the level of NT-ProBNP was significantly higher in the IVIG-resistance group than that in the IVIG-response group (2685 [551.50–7010.00] vs 975.00 [387.00–2560.00], P = 0.006). Similar findings were noted in the age subgroups except for patients older than 6 years. NT-ProBNP did not differ between the CAL (*n* = 45, median: 1070 pg/ml, IQR: 390.5–2895.0 pg/ml) and non-CAL group (*n* = 348, median: 1095 pg/ml, IQR: 405.0–2842.5 pg/ml) in all patients as well as in the age subgroups (all *P* > 0.05). The level of NT-ProBNP in the KDSS group [median: 24800 ng/ml, IQR (6500-35,004 pg/ml)] was significantly higher than that in the non-KDSS group [median: 1130 ng/ml, IQR (371-2740 pg/ml), *P* = 0.008].

**Table 2:** Comparison of N-terminal pro-brain natriuretic peptide level between IVIG-resistance and IVIG-response group stratified by age

	IVIG-resistance group	IVIG-response group	P
Overall (*n* = 393)	54	339	
Age (month)	28.50 [14.00–57.00]	24.00 [13.00–42.00]	0.051
NT-ProBNP	2685 [551.50–7010.00]	975.00 [387.00–2560.00]	0.006*
< 1 year (n = 79)	8	71	
Age (month)	7 [4–8]	7 [5–8]	0.941
NT-ProBNP	3950.00 [1745.00–6252.50]	1130.00[471.00–2790.00]	0.012*
1–2 years (n = 109)	14	95	
Age (month)	16.50 [13.75–20.25]	17.00 [14.00–20.00]	0.923
NT-ProBNP	2290.00 [494.75–5347.50]	1080.00 [472.00–2580.00]	0.001*
2–6 years (n = 176)	23	153	
Age (month)	39.00 [29.00–54.00]	38.00 [30.00–49.00]	0.491
NT-ProBNP	3770.00 [528.00–8800.00]	798.00 [305.00–2085.00]	< 0.001*
> 6 years (n = 29)	9	20	
Age (month)	83.00 [76.00–94.50]	86.00 [80.00–101.50]	0.308
NT-ProBNP	609.00 [207.00–9775.00]	2110.00 [369.75–9742.50]	0.822

The data are presented as the median with the 25th and 75th percentiles in square brackets for continuous variables

IVIG, intravenous immunoglobulin; NT-ProBNP, N-terminal probrain natriuretic peptide;

*Statistically significant (P < 0.05)

The cutoff value of NT-ProBNP for predicting IVIG resistance in all patients was 3755 pg/ml (area under the curve (AUC) = 0.64), with a sensitivity of 44.4%, a specificity of 84.1%, a positive predictive value (PPV) of 30.8%, a negative predictive value(NPV) of 90.5% and a diagnostic accuracy of 78.6%. The odds ratio (OR) of the cutoff value of NT-ProBNP was 4.22 (95% confidence interval (CI): 2.29–7.78, *P* < 0.001).

**Fig. 2:**
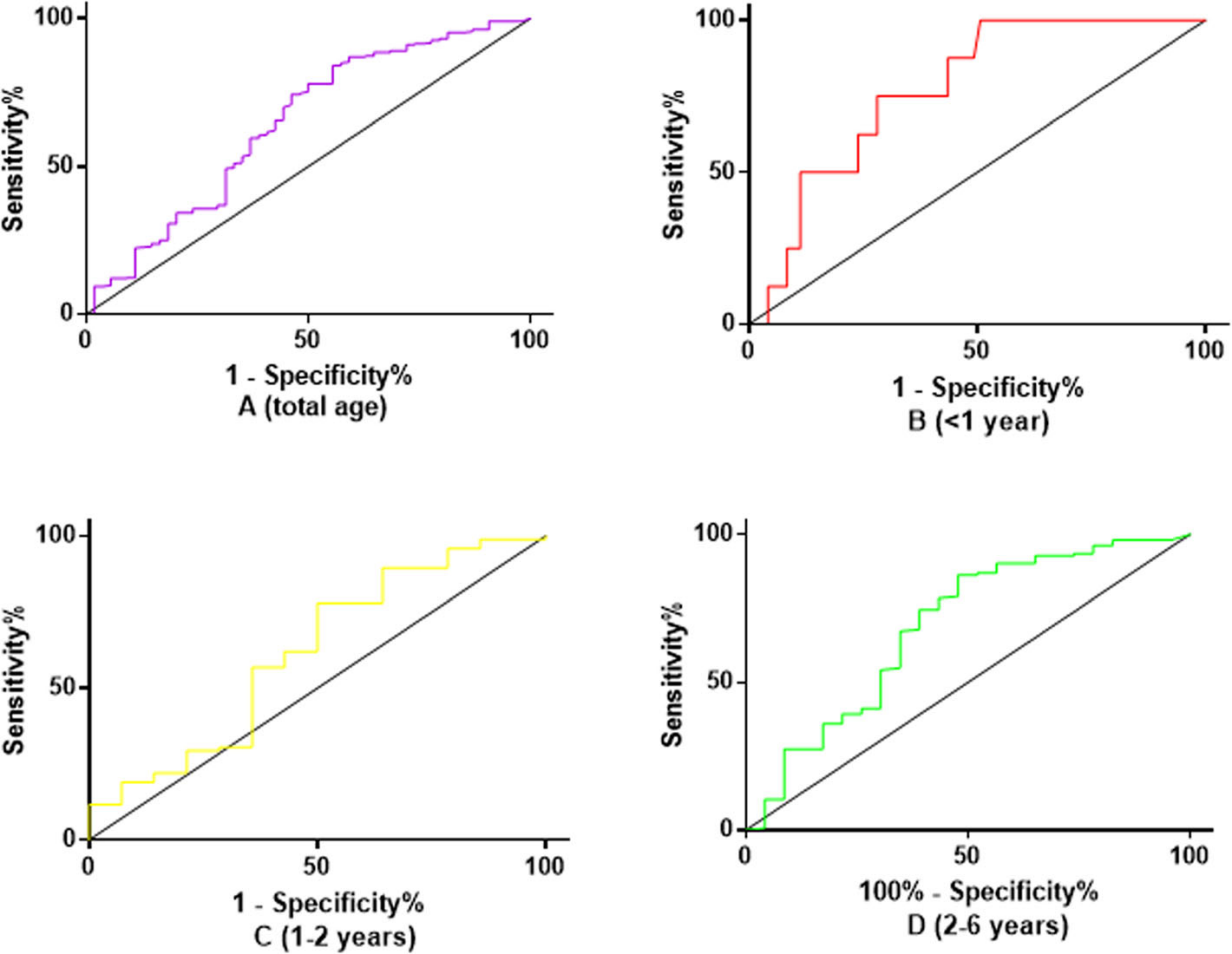
The receiver-operating-characteristic curve (ROC) for NT-ProBNP in IVIG resistance prediction among different age stratified group. **a** The ROC for NT-ProBNP in IVIG resistance prediction in total age. **b** The ROC for NT-ProBNP in IVIG resistance prediction in patients younger than 1 year old. **c** The ROC for NT-ProBNP in IVIG resistance prediction in patients aged 1–2 years. **d** The ROC for NT-ProBNP in IVIG resistance prediction in patients aged 2–6 years

The cutoff value in the group with patients younger than one year was 2480 pg/ml (AUC = 0.77), with a sensitivity of 75.0%, specificity of 71.8%, PPV of 23.1%, NPV of 96.2%, a diagnostic accuracy of 72.3%, and an OR of 7.65 (95% CI: 1.42–41.12, *P* = 0.014). The cutoff value in the group aged between 1 and 2 years old was 2800 pg/ml (AUC = 0.61), the sensitivity and specificity were 50.0 and 77.9%, respectively, and the PPV, NPV and diagnostic accuracy were 25.0, 91.4, and 74.3%, respectively. The OR of this cutoff value of NT-ProBNP was 3.52 (95% CI: 1.11–11.18, *P* = 0.045). The cutoff value in the group aged 2~6 years was 3710 pg/ml (AUC = 0.69), the sensitivity, specificity, PPV, NPV, and diagnostic accuracy were 52.2, 86.3, 36.4, 92.3, and 81.8%, respectively. The OR of the new cutoff value of NT-ProBNP was 6.86 (95% CI 2.68–17.53, *P* < 0.001) (Table 3 and Fig. 2). The diagnostic sensitivity and specificity according to ROC-optimized decision limits are shown in Table 4.

**Table 3:** Different cutoff values of N-terminal pro-brain natriuretic peptide in predicting IVIG resistance in KD stratified by age

Age group	Cutoff value of NT-ProBNP	Category	Response to IVIG		Sen	Spe	PPV	NPV	Diagnostic accuracy	OR (95%CI)	AUC	P
			Resistance	Response								
< 1 year	NT-ProBNP≥2480 pg/ml	High risk	6	20	75.0%	71.8%	23.1%	96.2%	72.3%	7.65 (1.42–41.12)	0.77	0.014*
		Low risk	2	51								
1–2 years	NT-ProBNP≥2800 pg/ml	High risk	7	21	50.0%	77.9%	25.0%	91.4%	74.3%	3.52 (1.11–11.18)	0.61	0.045*
		Low risk	7	74								
2–6 years	NT-ProBNP≥3710 pg/ml	High risk	12	21	52.2%	86.3%	36.4%	92.3%	81.8%	6.86 (2.68–17.53)	0.69	< 0.001*
		Low risk	11	132								
Total age	NT-ProBNP≥3755 pg/ml	High risk	24	54	44.4%	84.1%	30.8%	90.5%	78.6%	4.22 (2.29–7.78)	0.64	< 0.001*
		Low risk	30	285								

Sen, sensitivity; Spe, specificity; PPV, positive predictive value; NPV, negative predictive value; AUC, area under the curve;

NT-ProBNP, N-terminal probrain natriuretic peptide; IVIG, intravenous immunoglobulin;

*Statistically significant (P < 0.05)

**Table 4:** Diagnostic specificity and sensitivity according to receiver operating characteristic-optimized decision limits for N-terminal pro-B-type natriuretic peptide

	All (n = 393)		<1y (n = 79)		1-2y (n = 109)		2-6y (n = 176)	
Specificity (%)	Cut-point (pg/ml)	N	Cut-point (pg/ml)	N	Cut-point (pg/ml)	N	Cut-point (pg/ml)	N
95	9860.0	27	10,215.0	4	7060.0	4	12,150.0	13
99	32,900.0	4	19,250.0	1	12,300.0	1	32,900.0	4
Sensitivity (%)	Cut-point (pg/ml)	N	Cut-point (pg/ml)	N	Cut-point (pg/ml)	N	Cut-point (pg/ml)	N
95	161.5	357	249	71	213.5	100	60	174
99	56.5	391	96.5	78	69	108	50	176

## Discussion

In this prospective study, we could establish that serum levels of NT-proBNP were significantly elevated in the IVIG-resistance group as compared with the IVIG-response group in a Western Chinese population. However, NT-ProBNP may be not suitable as a single marker to accurately predict IVIG resistance in a clinical setting because of its low sensitivity of 44.4%, which was partially inconsistent with previous studies [14–17]. Additionally, the best cut-off value appeared to be higher. As shown in the Additional file 1, the differences in the median age of enrolled subjects, definition of IVIG resistance, incidence of IVIG resistance, initial therapy protocols, timing of serum NT-ProBNP test, assays of NT-ProBNP measurement and genetic backgrounds may contribute to the different findings in our study as compared to previous studies (see Additional file 1). Furthermore, different inclusion and exclusion criteria may also explain these variable findings. For instance, in the study by Kim et al. in Korea [15], patients who presented with CAL before the initial IVIG treatment were excluded. However, previous studies [24] [16] have shown that the inflammatory response is likely to be more severe in these patients and excluding them may, therefore, lead to lower NT-ProBNP levels. Given the sufficient number of patients and prospective approach, the findings in our report may be more conclusive.

Most importantly, the nature of the age-dependent change in NT-ProBNP levels was not considered by previous studies [14–17], possibly as a consequence of small sample sizes. Our study was the first to examine the effectiveness of age-specific NT-ProBNP cutoff levels to predict IVIG resistance in children with KD. Consistent with our hypothesis, it was found that the serum level of NT-ProBNP did not differ in KD patients older than 6 years, and the cutoff value of NT-ProBNP was also different in those aged < 1 year (2480 pg/ml), 1–2 years (2800 pg/ml) and 2–6 years (3710 pg/ml) compared to all KD patients (3755 pg/ml). In addition, after age-matched stratification, the sensitivity of the cutoff value was slightly higher in children aged 1–2 (50.0%) and 2–6 years (52.2%), and remarkably increased in children aged < 1 year (75.0%), while the specificity was still high in all three groups (71.8–86.3%).

These results suggest that applying a cutoff value of 3755 pg/ml to all KD children, particularly those younger than two years, would miss many IVIG non-responders. Therefore, our findings provide important evidence that it is more reasonable and precise to apply different cut-off values of NT-ProBNP based on age when aiming at predicting IVIG resistance in KD.

The prediction of IVIG resistance is one of the main clinical issues and, consequently, one of the most extensively studied topics in KD. Researchers have previously made attempts to find criteria and markers for such resistance. An elevation of serum neutrophils [25–28], CRP [25, 27, 29, 30], total bilirubin [27, 30], as well as a lower platelet count [25, 29, 31], hyponatremia [25, 27, 31], and hypoalbuminemia [26–28] are commonly observed in KD patients, which is similar to our findings. Several scoring systems incoporating these biomarkers, such as the Kobayashi [25], Egami [29] and Sano [30] system, have been used to identify IVIG resistance in KD in Japan. However, they seemed to be of less clinical relevance in non-Japanese populations such as those in the US [32, 33], Korea [34], Germany [35], Spain [36] and China [37, 38]. Recently, several predictive Chinese models including the ones by Formosa [26], Yang [27], Tang [28] and Hua [31] have been developed. They also showed variable predictive effectiveness even within China [37, 38]. We had previously tested the predictive value of all these risk-scoring systems in our population. As shown in the Additional file 2, we had found that the Kobayashi, Egami, Sano, Yang’s, and Hua’s score had a relatively high specificity of 78.8–94.1%, but an extremely low sensitivity of 16.7–35.2% (see Additional file 2). The performance of Formosa’s and Tang’s systems also showed only moderate sensitivity (57.4–61.1%) and specificity (54.0–67.3%). In the present study, the predictive value of NT-ProBNP as a single marker for IVIG resistance seems to be comparable or slightly better compared to that of Formosa’s and Tang’s system in our population, although the sensitivity was also low (44.4%). However, after age-stratification, the sensitivity was slightly higher in patients aged 1–2 (50.0%) and 2–6 years (52.2%), and remarkably increased in patients aged < 1 year (75.0%), while the specificity was still high in these three groups (71.8–86.3%).

On the basis of these findings, it is evident that we can not identify all IVIG non-responders using any of the above risk scores, including NT-ProBNP. However, as a parameter obtained from routine blood tests, NT-ProBNP appears to be a cost-effective alternative that may provide additional information on IVIG resistance, particularly in children under one year. Moreover, unlike the aforementioned risk-scoring systems, the predictive value of the NT-ProBNP seems to be more consistent and stable among different populations despite different varied cut-off values, sensitivities and specificities. Nevertheless, given the unknown origin of KD and the above findings, we suggest that a prediction model combining NT-ProBNP with other specific indicators might have a better performance.

This study has some potential limitations. Firstly, this study was performed in a single institution. Our hospital is the largest children medical center in Southwest China, which may lead to a selection bias in that more severely ill patients are being admitted to us. Secondly, the present study was a prospective cohort study and had strict inclusion and exclusion criteria. The findings in our study are therefore only applicable to Chinese KD patients receiving the standardized IVIG treatment (2 g/kg) within ten days from fever onset. Finally, the age stratification was based on a previous study and age-matched healthy children were not enrolled to determine the reference valus of NT-ProBNP. However, studies have proven a significant negative correlation between age and plasma levels of NT-ProBNP in children. Additionally, previous multicenter studies from both the United States [39] and Europe [40] have revealed that NT-ProBNP measurements obtained with the same assay but at different study sites are highly comparable both for physiological and pathological plasma samples. More importantly, the main objective of our study was to determine the effectiveness of NT-ProBNP in the prediction of IVIG resistance. Therefore, this limitation may not affect our main findings.

Despite these limitations, this study is the first to determin the usefulness of age-specific NT-proBNP cutoff levels for the prediction of IVIG resistance in a prospective study with a relatively large sample size. We found that NT-proBNP is a complementary laboratory marker for the prediction of IVIG resistance in KD, particular in children younger than one year. Larger prospective multicenter studies with standardized therapy protocols are warranted to investigate the usefulness of age- specific NT-proBNP cutoff values, either in an algorithm or in combination with other clinical criteria and laboratory values, which will likely increase its sensitivity in predicting IVIG resistance in KD patients.

## Conclusions

NT-proBNP is a complementary laboratory marker for the prediction of IVIG resistance. The application of age-specific cutoff values for NT-ProBNP increases its ability to predict IVIG resistance in KD.

## Supplementary information

**Additional file 1.** Comparison of studies with respect to the effectiveness of NT-ProBNP for IVIG resistance prediction in KD.

**Additional file 2.** The sensitivity, specificity, PPV and NPV of all avaliable risk-scoring systems for IVIG resistance prediction in our population.

## Data Availability

All data generated or analyzed during this study are included in this published article and the supplementary files.

## Abbreviations

AUC: area under the curve
CAL: coronary artery lesion
CI: confidence interval
CRP: C-reactive protein
IVIG: intravenous immunoglobulin
KD: Kawasaki Disease
KDSS: Kawasaki Disease shock syndrome
NPV: negative predictive value
NT-ProBNP: N-terminal pro-brain natriuretic peptide
OR: odds ratio
PPV: positive predictive value
ROC: receiver operating characteristic

## References

[1] Uehara R, Belay ED, Maddox RA, Holman RC, Nakamura Y, Yashiro M (2008). Analysis of potential risk factors associated with nonresponse to initial intravenous immunoglobulin treatment among Kawasaki disease patients in Japan. Pediatr Infect Dis J.

[2] Kobayashi T, Saji T, Otani T, Takeuchi K, Nakamura T, Arakawa H (2012). Efficacy of immunoglobulin plus prednisolone for prevention of coronary artery abnormalities in severe Kawasaki disease (RAISE study): a randomised, open-label, blinded-endpoints trial. Lancet..

[3] Burns JC, Best BM, Mejias A, Mahony L, Fixler DE, Jafri HS (2008). Infliximab treatment of intravenous immunoglobulin-resistant Kawasaki disease. J Pediatr.

[4] Maggio MC, Cimaz R, Alaimo A, Comparato C, Di Lisi D, Corsello G (2019). Kawasaki disease triggered by parvovirus infection: an atypical case report of two siblings. J Med Case Rep.

[5] Kone-Paut I, Cimaz R, Herberg J, Bates O, Carbasse A, Saulnier JP (2018). The use of interleukin 1 receptor antagonist (anakinra) in Kawasaki disease: a retrospective cases series. Autoimmun Rev.

[6] Hamada H, Suzuki H, Onouchi Y, Ebata R, Terai M, Fuse S (2019). Efficacy of primary treatment with immunoglobulin plus ciclosporin for prevention of coronary artery abnormalities in patients with Kawasaki disease predicted to be at increased risk of non-response to intravenous immunoglobulin (KAICA): a randomised controlled, open-label, blinded-endpoints, phase 3 trial. Lancet..

[7] Dahdah N, Siles A, Fournier A, Cousineau J, Delvin E, Saint-Cyr C (2009). Natriuretic peptide as an adjunctive diagnostic test in the acute phase of Kawasaki disease. Pediatr Cardiol.

[8] Lemos JA, De MDK, Drazner MH (2003). B-type natriuretic peptide in cardiovascular disease. Lancet..

[9] Morita E, Yasue H, Yoshimura M, Ogawa H, Jougasaki M, Matsumura T (1993). Increased plasma levels of brain natriuretic peptide in patients with acute myocardial infarction. Circulation..

[10] Lindholm D, James SK, Gabrysch K, Storey RF, Himmelmann A, Cannon CP (2018). Association of Multiple Biomarkers with Risk of all-cause and cause-specific mortality after acute coronary syndromes: a secondary analysis of the PLATO biomarker study. JAMA Cardiol.

[11] Wolsk E, Claggett B, Pfeffer MA, Diaz R, Dickstein K, Gerstein HC, et al. Role of B-type natriuretic peptide and N-terminal prohormone BNP as predictors of cardiovascular morbidity and mortality in patients with a recent coronary event and type 2 diabetes mellitus. J Am Heart Assoc. 2017;6(6). 10.1161/JAHA.116.004743 PMC5669146 28554908

[12] Brozaitiene J, Mickuviene N, Podlipskyte A, Burkauskas J, Bunevicius R (2016). Relationship and prognostic importance of thyroid hormone and N-terminal pro-B-type natriuretic peptide for patients after acute coronary syndromes: a longitudinal observational study. BMC Cardiovasc Disord.

[13] Siva Sankara C, Rajasekhar D, Vanajakshamma V, Praveen Kumar BS, Vamsidhar A (2015). Prognostic significance of NT-proBNP, 3D LA volume and LV dyssynchrony in patients with acute STEMI undergoing primary percutaneous intervention. Indian Heart J.

[14] Kim SY, Han MY, Cha SH, Jeon YB (2013). N-terminal pro-brain natriuretic peptide (NT proBNP) as a predictive indicator of initial intravenous immunoglobulin treatment failure in children with Kawasaki disease: a retrospective study. Pediatr Cardiol.

[15] Kim HK, Oh J, Hong YM, Sohn S (2011). Parameters to guide retreatment after initial intravenous immunoglobulin therapy in Kawasaki disease. Korean Circ J..

[16] Yoshimura K, Kimata T, Mine K, Uchiyama T, Tsuji S, Kaneko K (2013). N-terminal pro-brain natriuretic peptide and risk of coronary artery lesions and resistance to intravenous immunoglobulin in Kawasaki disease. J Pediatr.

[17] Lee HY, Song MS (2016). Predictive factors of resistance to intravenous immunoglobulin and coronary artery lesions in Kawasaki disease. Korean J Pediatr.

[18] Nir A, Lindinger A, Rauh M, Bar-Oz B, Laer S, Schwachtgen L (2009). NT-pro-B-type natriuretic peptide in infants and children: reference values based on combined data from four studies. Pediatr Cardiol.

[19] Lin CW, Zeng XL, Zhang JF, Meng XH (2014). Determining the optimal cutoff values of plasma N-terminal pro-B-type natriuretic peptide levels for the diagnosis of heart failure in children of age up to 14 years. J Card Fail.

[20] Li S, Xiao Z, Li L, Hu B, Zhou Z, Yi S (2018). Establishment of normal reference values of NT-proBNP and its application in diagnosing acute heart failure in children with severe hand food and mouth disease. Medicine (Baltimore).

[21] Schwachtgen L, Herrmann M, Georg T, Schwarz P, Marx N, Lindinger A (2005). Reference values of NT-proBNP serum concentrations in the umbilical cord blood and in healthy neonates and children. Z Kardiol.

[22] Newburger JW, Takahashi M, Gerber MA, Gewitz MH, Tani LY, Burns JC (2004). Diagnosis, treatment, and long-term management of Kawasaki disease: a statement for health professionals from the committee on rheumatic fever, endocarditis, and Kawasaki disease, council on cardiovascular disease in the young, American Heart Association. Pediatrics..

[23] Arjunan K, Daniels SR, Meyer RA, Schwartz DC, Barron H, Kaplan S (1986). Coronary artery caliber in normal children and patients with Kawasaki disease but without aneurysms: an echocardiographic and angiographic study. J Am Coll Cardiol.

[24] Jone PN, Anderson MS, Mulvahill MJ, Heizer H, Glode MP, Dominguez SR (2018). Infliximab plus intravenous immunoglobulin (IVIG) versus IVIG alone as initial therapy in children with Kawasaki disease presenting with coronary artery lesions: is dual therapy more effective?. Pediatr Infect Dis J.

[25] Kobayashi T, Inoue Y, Takeuchi K, Okada Y, Tamura K, Tomomasa T (2006). Prediction of intravenous immunoglobulin unresponsiveness in patients with Kawasaki disease. Circulation..

[26] Lin MT, Chang CH, Sun LC, Liu HM, Chang HW, Chen CA (2016). Risk factors and derived Formosa score forintravenous immunoglobulin unresponsiveness in Taiwanese children with Kawasaki disease. J Formos Med Assoc.

[27] Yang S, Song R, Zhang J, Li X, Li C (2019). Predictive tool for intravenous immunoglobulin resistance of Kawasaki disease in Beijing. Arch Dis Child.

[28] Tang Y, Yan W, Sun L, Huang J, Qian W, Ding Y (2016). Prediction of intravenous immunoglobulin resistance in Kawasaki disease in an East China population. Clin Rheumatol.

[29] Egami K, Muta H, Ishii M, Suda K, Sugahara Y, Iemura M (2006). Prediction of resistance to intravenous immunoglobulin treatment in patients with Kawasaki disease. J Pediatr.

[30] Sano T, Kurotobi S, Matsuzaki K, Yamamoto T, Maki I, Miki K (2007). Prediction of non-responsiveness to standard high-dose gamma-globulin therapy in patients with acute Kawasaki disease before starting initial treatment. Eur J Pediatr.

[31] Hua W, Sun Y, Wang Y, Fu S, Wang W, Xie C (2017). A new model to predict intravenous immunoglobin-resistant Kawasaki disease. Oncotarget..

[32] Sleeper LA, Minich LL, McCrindle BM, Li JS, Mason W, Colan SD (2011). Evaluation of Kawasaki disease risk-scoring systems for intravenous immunoglobulin resistance. J Pediatr.

[33] Loomba RS, Raskin A, Gudausky TM, Kirkpatrick E (2016). Role of the Egami score in predicting intravenous immunoglobulin resistance in Kawasaki disease among different ethnicities. Am J Ther.

[34] Kim BY, Kim D, Kim YH, Ryoo E, Sun YH, Jeon IS (2016). Non-responders to intravenous immunoglobulin and coronary artery dilatation in Kawasaki disease: predictive parameters in Korean children. Korean Circ J.

[35] Jakob A, von Kries R, Horstmann J, Hufnagel M, Stiller B, Berner R (2018). Failure to predict high-risk Kawasaki disease patients in a population-based study cohort in Germany. Pediatr Infect Dis J.

[36] Sanchez-Manubens J, Anton J, Bou R, Iglesias E, Calzada-Hernandez J, Borlan S (2016). Role of the Egami score to predict immunoglobulin resistance in Kawasaki disease among a Western Mediterranean population. Rheumatol Int.

[37] Song R, Yao W, Li X (2017). Efficacy of four scoring Systems in Predicting Intravenous Immunoglobulin Resistance in children with Kawasaki disease in a Children's Hospital in Beijing, North China. J Pediatr.

[38] Qian W, Tang Y, Yan W, Sun L, Lv H (2018). A comparison of efficacy of six prediction models for intravenous immunoglobulin resistance in Kawasaki disease. Ital J Pediatr.

[39] Sokoll LJ, Baum H, Collinson PO, Gurr E, Haass M, Luthe H (2004). Multicenter analytical performance evaluation of the Elecsys proBNP assay. Clin Chem Lab Med.

[40] Collinson PO, Barnes SC, Gaze DC, Galasko G, Lahiri A, Senior R (2004). Analytical performance of the N terminal pro B type natriuretic peptide (NT-proBNP) assay on the Elecsys 1010 and 2010 analysers. Eur J Heart Fail.

