# Supplementary material for: The role of age-specific N-terminal pro-brain natriuretic peptide cutoff values in predicting intravenous immunoglobulin resistance in Kawasaki disease: a prospective cohort study
Source: Pediatr Rheumatol Online J. 2019 Sep 18;17:65. doi: 10.1186/s12969-019-0368-8 (PMC6751871; doi:10.1186/s12969-019-0368-8)
Supplement: Supplementary file 1 — Additional file 1. Comparison of studies with respect to the effectiveness of NT-ProBNP for IVIG resistance prediction in KD. [file 12969_2019_368_MOESM1_ESM.docx]

**Additional file 1**. Comparison of studies with respect to the effectiveness of NT-ProBNP for IVIG resistance prediction in KD

|  | This study | Hyun Kwon Kim | Ken Yoshimura | So Youn Kim | Hye Young Lee |
| --- | --- | --- | --- | --- | --- |
| Publication year | 2019 | 2011 | 2013 | 2013 | 2016 |
| Country | China | Korea | Japan | Korea | Korea |
| Study duration | 2015-2018 | 2008-2010 | 2008-2012 | 2003-2011 | 2010-2015 |
| Research design | Prospective | Prospective | Prospective | Retrospective | Retrospective |
| Sample size (n) | 393 | 129 | 80 | 135 | 287 |
| Age (months) | 1-125 | 29.0±20.8 | 2-76 | <60 | 2-159 |
| Sampling days from fever onset (d) | 5.0 (4.0-5.0) | Unclear | 4.0 (3.0-6.0) | Unclear | Unclear |
| Exclusion criteria | Yes ^a^ | Yes ^b^ | Yes ^c^ | Yes ^d^ | Unclear |
| Definition of IVIG resistance ^e^ | ≥36h | ≥48h | ≥24h | ≥36h | ≥36h |
| IVIG resistance, (%) | 13.7% | 17.0% | 21.3% | 16.3% | 11.8% |
| Initial IVIG dosage | 2g/Kg | 2g/Kg | 2g/Kg | 2g/Kg | 2g/Kg |
| Aspirin dosage (mg/kg/d) | 30-50 | 80-100 | 30 | 50 | 50 |
| Detection assay for NT-ProBNP | Roche | Unclear | Roche | Roche | Unclear |
| Cutoff of NT-ProBNP (pg/ml) | ≥3755 | ≥479 | ≥800 | ≥1093 | ≥628.6 |
| Sensitivity | 44.4% | 78.9% | 71.0% | 70.0% | 78.8% |
| Specificity | 84.1% | 86.0% | 62.0% | 76.5% | 58.2% |
| PPV | 30.8% | - | - | - | - |
| NPV | 90.5% | - | - | - | - |

IVIG: intravenous immunoglobulin, NT-pro-BNP: N-terminal Pro-brain Natriuretic Peptide

PPV: positive predictive value, NPV: negative predictive value

^a^ : Patients who received IVIG therapy in other medical facilities, or earlier than 4 or later than 10 days from fever onset, or lacked laboratory data or follow-up results.

^b^ : Patients who presented with CAL before initial IVIG treatment was excluded.

^c^ : The presence of another disease known to mimic KD, previous diagnosis of KD, and incomplete KD.

^d^ : Previous diagnosis of KD and any patients admitted to hospital in a subacute phase of KD after undergoing initial treatment at other hospitals.

^e^ : The definition of IVIG resistance refers to the fever duration after initial IVIG treatment.
