# Supplementary material for: The role of age-specific N-terminal pro-brain natriuretic peptide cutoff values in predicting intravenous immunoglobulin resistance in Kawasaki disease: a prospective cohort study
Source: Pediatr Rheumatol Online J. 2019 Sep 18;17:65. doi: 10.1186/s12969-019-0368-8 (PMC6751871; doi:10.1186/s12969-019-0368-8)
Supplement: Supplementary file 2 — Additional file 2. The sensitivity, specificity, PPV and NPV of all avaliable risk-scoring systems for IVIG resistance prediction in our population. [file 12969_2019_368_MOESM2_ESM.docx]

**Additional file 2.** The sensitivity, specificity, PPV and NPV of all avaliable risk-scoring systems for IVIG resistance prediction in our population

| Risk scoring-systems | Sensitivity(%) | Specificity(%) | PPV(%) | NPV(%) | Diagnostic accuracy(%) |
| --- | --- | --- | --- | --- | --- |
| Kobayashi | 35.2 | 84.4 | 26.4 | 89.1 | 77.6 |
| Egami | 31.5 | 78.8 | 19.1 | 87.8 | 72.3 |
| Sano | 16.7 | 94.1 | 31.0 | 87.6 | 83.5 |
| Formosa | 61.1 | 54.0 | 17.5 | 89.7 | 55.0 |
| Tang’s | 57.4 | 67.3 | 21.8 | 90.8 | 65.9 |
| Yang’s | 42.6 | 79.1 | 24.5 | 89.6 | 74.0 |
| Hua’s | 27.8 | 83.5 | 21.1 | 87.9 | 75.8 |

PPV: positive predictive value, NPV: negative predictive value
